# Clinical Genetic and Genomic Testing in Congenital Heart Disease and Cardiomyopathy

**DOI:** 10.3390/jcm13092544

**Published:** 2024-04-26

**Authors:** Mahati Pidaparti, Gabrielle C. Geddes, Matthew D. Durbin

**Affiliations:** 1 Division of Neonatal-Perinatal Medicine, Department of Pediatrics, Indiana University School of Medicine, Indianapolis, IN 46202, USA; mpidapar@iu.edu; 2 Department of Medical and Molecular Genetics, Indiana University School of Medicine, Indianapolis, IN 46202, USA; egeddes@iu.edu; 3 Herman B Wells Center for Pediatric Research, 1044 W. Walnut, Indianapolis, IN 46202, USA

**Keywords:** congenital heart disease 1, cardiomyopathy 2, genetic testing 3, genomic testing, personalized medicine, genome sequencing

## Abstract

Congenital heart disease (CHD) and cardiomyopathies are the leading cause of morbidity and mortality worldwide. These conditions are often caused by genetic factors, and recent research has shown that genetic and genomic testing can provide valuable information for patient care. By identifying genetic causes, healthcare providers can screen for other related health conditions, offer early interventions, estimate prognosis, select appropriate treatments, and assess the risk for family members. Genetic and genomic testing is now the standard of care in patients with CHD and cardiomyopathy. However, rapid advances in technology and greater availability of testing options have led to changes in recommendations for the most appropriate testing method. Several recent studies have investigated the utility of genetic testing in this changing landscape. This review summarizes the literature surrounding the clinical utility of genetic evaluation in patients with CHD and cardiomyopathy.

## 1. Introduction

Congenital heart disease (CHD) and cardiomyopathies are significant causes of morbidity and mortality worldwide [1,2]. Both CHD and cardiomyopathies have high rates of clinically detectable genetic causes, including chromosomal anomalies, copy number variants, and monogenic disruptions [2,3,4,5,6,7]. Genetic testing has been recommended for several years to identify these genetic causes [8,9]. There are many reasons to obtain genetic testing in search of a genetic diagnosis [1,10]. Obtaining a genetic diagnosis can help in reproductive planning, provide valuable information about the presence of comorbidities in other organ systems, and help identify conditions that require increased screening and early intervention services. A genetic diagnosis can also inform prognosis and help guide the selection of appropriate therapies. Furthermore, a genetic diagnosis may be relevant to both a patient and their family members [11,12,13]. 

Around 25–50% of patients with CHD and cardiomyopathies have abnormal genetic testing results [14,15,16,17]. Despite recommendations for genetics evaluation, genetic testing is underutilized and highly variable. A recent multicenter cohort study revealed that only 55% of newborns with surgical CHD underwent genetic testing, with significant variability across institutions (42–78%) [18]. This was concerning, given that testing had a high diagnostic yield of 44% [18]. However, a follow-up analysis of a subset of the population demonstrated that implementing genetic testing guidelines led to an increase in the testing rate and resulted in a better diagnostic yield using newer modalities [19]. There is a clear need to promote knowledge and develop standardized approaches to genetic testing to benefit patients with CHD and cardiomyopathy [15,19,20,21]. Since there are multiple recent reviews of CHD and cardiomyopathy etiology [2,6,7,22,23,24,25], this review focuses specifically on clinical genetic and genomic testing for patients with CHD and cardiomyopathy. 

## 2. Background

CHD is the most common birth defect and the leading cause of death due to birth defects [26,27,28,29,30,31,32]. Around one-third of CHD requires surgery within the first year of life [26,27,28,29,30,31,32]. Cardiomyopathies cause similar disease burdens for both children and adults and often lead to heart transplantation [2,33,34]. The prevalence estimates range from up to 1 per 100 in CHD, to 1 of 250 in dilated cardiomyopathy (DCM) [2,35], and 1 per 500 in hypertrophic cardiomyopathy (HCM). While we previously thought that CHD and cardiomyopathies were separate entities, the overlap between them has become clearer as our understanding of genetics expands [2,35]. 

CHD and cardiomyopathies may occur independently or in combination with other systemic issues, like extracardiac congenital malformations, neuromuscular diseases, and inborn errors of metabolism [1,2,4,7,35,36,37,38,39]. Hence, both CHD and cardiomyopathies can be classified as either isolated or syndromic [4,37]. If a patient has other extracardiac health issues or anomalies, they are considered to have syndromic CHD/cardiomyopathy, whereas if they do not have any other related health concerns, they are considered to have isolated CHD/cardiomyopathy.

Classification systems have been developed for CHD based on the specific anatomy of the heart [40]. Cardiomyopathies can be classified into three main categories based on specific imaging and functional features: HCM, DCM, or restrictive cardiomyopathy (RCM). Depending on the source of information, two additional categories may or may not be included, which are left ventricular noncompaction cardiomyopathy (LVNC) and arrhythmogenic right ventricular cardiomyopathy (ARVC). Each of these forms of CHD and cardiomyopathy has its own set of core genes that have been identified as pathogenic, though there is significant overlap between groups [17,35]. 

## 3. Common Clinical Genetic and Genomic Tests

Genetic testing is recommended to identify the cause of CHD and cardiomyopathies [8,9]. Clinical assessment and differential diagnoses help determine the most effective testing modalities for specific patients, though these may need to be adjusted based on the availability of institutional resources. As testing methods continue to evolve, it is often helpful to be familiar with local resources, including the availability of a medical geneticist, a genetic counselor, or a laboratory geneticist. They can provide helpful insights and answer questions about testing options for specific cases. To begin to make an informed decision, it is crucial to understand the benefits and limitations of different genetic testing modalities.

### 3.1. Chromosome Analysis

Early clinical genetic evaluation methods involved visibly evaluating the chromosomes under magnification, and some version of this method is still utilized clinically today. Large chromosomal anomalies include aneuploidies, very large deletions or duplications, inversions, and translocations. They can be detected by chromosome analysis or karyotype analysis. In 1959–1960, descriptions of patients with underlying chromosomal anomalies, such as trisomy 18, trisomy 21, and monosomy X, were published [41]. These chromosomal anomalies are a well-described and frequent etiology of CHD, accounting for up to 8–12% of cases, with trisomy 21 being the most common diagnosis [1,23]. These diagnoses are also occasionally associated with cardiomyopathies [35].

Karyotypes are useful in patients with CHD or cardiomyopathy when there is a concern about an aneuploidy. They are usually part of the initial assessment when there is a suspicion of trisomy 21. In such cases, karyotyping is used to detect 3–4% of cases caused by translocations [42]. This is because karyotyping can detect balanced translocations and inversions, which are not currently detectable with any other test. In karyotype analysis, the source of the testing sample needs to be considered since karyotypes need to be performed on actively dividing cells, so they cannot be performed on buccal samples or extracted DNA. 

Advances in karyotype culture techniques and utilization of stains have improved resolution, allowing the clear identification of individual chromosomes and specific regions within the chromosomes [41,43]. This allowed the progressive identification of smaller areas of deletions or duplications, termed copy number variants (CNVs). In 1981, multiple reports led to the initial proposal that 22q11 was the causative area for the phenotype associated with 22q11.2 deletion syndrome [44]. Once a candidate area was identified, this finding was quickly replicated and published in 1982 [45]. Currently, the 22q11.2 deletion syndrome is understood to be the most common CNV identified in patients with CHD [23]. There are also rare reports of cardiomyopathy in 22q11.2 deletion, though they are mostly in association with a CHD [35]. 

### 3.2. Chromosomal Microarray

Chromosomal microarray (CMA) is a technology that measures the hybridization of a patient’s sample to a surface. It can detect CNVs much smaller than karyotype [43]. The clinical use of CMAs became more common in the 2000s and increased significantly after 2010 [43]. This was when the American Journal of Human Genetics published a statement recommending CMA as the first-tier evaluation for individuals with unexplained developmental delays, intellectual disabilities, autism spectrum disorders, or multiple congenital anomalies, including CHD and cardiomyopathies [43,46]. CMAs can detect many CNVs commonly associated with CHD and cardiomyopathies, including 22q11.2 deletion syndrome, as well as both 1p36 deletion, and 6q25.1 deletion, which are associated with CHD and cardiomyopathy [23,35]. The clinical use of CMA for CHD patients is highly variable [18]. Many centers use CMA as a standard genetic screening tool, while others still do not have the infrastructure to complete routine CMA. In recent years, many centers have also moved to genomic testing, which is now the standard of care [21]. 

### 3.3. Targeted Gene Sequencing/Genetic Testing Panels

A significant breakthrough in genetic testing occurred with the development of DNA sequencing techniques in 1977. This innovation enabled the identification of single nucleotide changes [47,48]. Since then, sequencing technologies evolved, and targeted sequencing is now commercially available to identify single nucleotide genetic alterations in specific genes or entire panels of genes that are implicated in CHD and cardiomyopathies. Targeted genetic testing provides greater sequencing coverage for the target gene and may detect variants that broader genomic testing may miss.

When completing or reviewing gene panel testing, it is important to be familiar with the limitations. For example, while most modern genetic testing panels include both sequencing and deletion/duplication analysis, many older modalities do not include these features, leading to the missed detection of these variants. For example, some genetic panels may not detect a common variant in MYBPC3 that can cause cardiomyopathy. This variant is located in deep intronic regions, which may not be covered by panels that only sequence exonic regions [49]. In order for a clinician to determine if genetic testing is appropriate, they need to review testing reports to understand the exact methods used. As genetic testing techniques vary and evolve over time, it is crucial to obtain the original genetic testing report for review both during the evaluation and in the future.

### 3.4. Genomic Testing with Exome Sequencing 

The Human Genome Project was started in 1990 with a “first draft” of the human genome developed in 2001 and completed in 2003 [50,51]. During this period, sequencing technologies improved with the development of more efficient techniques known as “next-generation sequencing” [47]. The first commercial release of a next-generation sequencing platform occurred in 2005, making genome sequencing research accessible by the late 2000s [47]. Initially, clinical next-generation sequencing was used for selected panels of genes associated with diseases. In October 2011, clinical exome sequencing became available [52]. By 2021, the American College of Medical Genetics and Genomics updated recommendations to first-tier testing with exome sequencing or genome sequencing for all individuals with developmental delay, intellectual disability, or congenital anomalies, like CHD [53]. 

Exome sequencing captures exons, or the ~1–2% of nuclear DNA that is protein-coding, representing more than 20,000 genes. Exome sequencing has demonstrated utility in CHD. One study, which analyzed 60 known CHD genes, identified causative variants in up to 33% of familial CHD cases, leaving the opportunity for an even greater yield with expansion beyond these 60 genes [54]. However, exome sequencing has some limitations. One of the primary differences between exome and genome sequencing is that exome sequencing undergoes a step of exon capture before sequencing, to enrich the sample for coding portions of the DNA, and this process can introduce bias [55]. The ultimate coverage of coding regions in exome sequencing is incomplete, and the efficiency of covered regions depends on factors such as the type of capture used, bioinformatic mapping techniques, and sample quality [55]. Some of these limitations are overcome with genome sequencing.

### 3.5. Genomic Testing with Genome Sequencing

While exome sequencing focuses on the ~1–2% of DNA that is protein-coding, genome sequencing captures most of the nuclear DNA. This includes transcription enhancers and promoters outside of the exon that can be involved in CHD pathogenesis. Genome sequencing can also identify CNVs, certain structural variants, and intronic variants not detectable by exome sequencing [55]. Genome sequencing does not undergo exon capture, and as a result, the coverage of genome sequencing is more uniformly consistent [56]. 

Recent studies have demonstrated the utility of genome sequencing in patients with CHD and cardiomyopathy. In these studies, rapid genome sequencing in select cohorts of CHD patients identified clinically actionable results, in 27–46% of patients, and surpassed CMA in head-to-head comparisons [57,58,59]. However, other studies highlight limitations, including the burden of interpreting variants of uncertain significance, with differences in variant interpretation up to 43% [60]. Furthermore, while the coverage of genome sequencing is superior to exome sequencing, there are still gaps in genome sequencing coverage [55,61].

### 3.6. Mitochondrial Genome Sequencing

Variants in the mitochondrial genome are an important consideration for cardiomyopathies, but sequencing the mitochondrial genome presents unique challenges. Exome sequencing typically excludes the mitochondrial genome, and genome sequencing may exclude it, as well. Additionally, the burden of pathogenic mitochondrial variants may differ depending on cell type due to heteroplasmy, as mitochondrial populations segregate independently of nuclear genetic elements during cell division [62]. For this reason, to diagnose mitochondrial disease appropriately, affected tissue samples, such as skeletal muscle or the liver, may be required [62]. Furthermore, different heteroplasmy levels of the same variant may be associated with different mitochondrial disease presentations and ages of onset [62]. 

## 4. Current Limitations

It is important to consider certain limitations when conducting clinical genetic testing. One common obstacle is the lack of understanding of genetic diseases. For example, some genes, such as *SNIP1* and *SHROOM3*, have only recently been linked to cardiac disease; so, prior to this, pathogenic variants in these genes may have been unreported or considered incidental findings in patients undergoing testing for cardiac indications [63,64,65,66]. 

Another significant challenge is identifying variants of uncertain significance. This started with the increased utilization of CMA testing, and has become more difficult with the introduction of clinical genomic testing [41]. Although rare variants can be detected, it is often difficult to determine their clinical significance, if any [67]. As testing volumes increase, artificial intelligence techniques are being used to analyze the thousands of rare variants captured in testing [68]. This includes using natural language processing to search the medical literature for reports of overlapping phenotypes or patients with similar variants [68]. Still, variants of uncertain significance remain a major clinical challenge in genetic and genomic testing.

For effective genomic testing, a review of a patient’s complete medical history, along with a review of the current medical literature, is often necessary. In the future, additional basic, translational, and clinical research is needed.

## 5. Future Directions

Sequencing technology continues to advance, enabling progress in “long-read” sequencing [48,61,69,70]. Current clinical genomic testing techniques are limited to the “short-read” sequencing of DNA fragments with fewer than 300 base pairs [56,61,70]. These segments are amplified and then mapped to areas of the reference sequence that have overlapping sequence characteristics [56]. The resulting variants are then annotated and filtered for interpretation [56]. However, short-read genomic testing is limited in its ability to detect genetic anomalies due to the number of repetitive regions across the human genome [61]. Previous assessments have suggested that at least 4264 exons across 619 clinically relevant genes cannot be assessed by short-read genomic testing [61,71]. Repeat expansion disorders may also not be detectable on short-read genomic testing. An example relevant to cardiomyopathy is Friedreich’s ataxia, which is most commonly due to a biallelic intronic repeat expansion [72]. 

On the other hand, long-read sequencing can sequence DNA fragments from 10 kilobases to several megabases [48,61,69,70]. This means that long-read sequencing has the potential to identify complex structural rearrangements, such as translocations, inversions, and repeat expansion disorders [48,61,70]. In 2020, long-read sequencing facilitated the first complete sequencing of a chromosome, in which the telomere-to-telomere sequence of the X chromosome in a haploid cell line was published [73]. 

Additionally, long-read sequencing can identify methylation anomalies [70]. Diagnosing methylation disorders is important in the management of CHD and cardiomyopathies. For example, up to 20% of patients with Beckwith–Wiedemann syndrome have CHD, and the majority of patients can only be diagnosed with methylation testing [74]. Currently, diagnosing disorders with abnormal methylation patterns requires very specific clinical testing. Long-read genome sequencing represents a significant diagnostic advancement. However, long-read genome sequencing is not currently available in the clinical setting, and the timeline for when it will be accessible is unclear. 

Genetic testing has significant implications for patients with CHD and cardiomyopathy and their family members, as genetic diagnosis is becoming increasingly important in determining prognosis and guiding clinical care [11,12,13]. Several studies have linked a genetic diagnosis to postoperative outcomes in CHD [75,76]. In cardiomyopathy, several studies have linked genotype status to higher rates of heart failure events and worse outcomes [77,78]. Testing results can also influence clinical management; for example, certain high-risk genetic variants may, depending on clinical severity, prompt earlier discussions of cardioverter-defibrillator implantation [79]. Researchers continue to assess the role of pathogenic variants to determine risk stratification and prognostication. This will remain a work in progress as we discover more genetic variants associated with CHD and cardiomyopathies.

Many of the genetic disorders common in patients with CHD and cardiomyopathy have specific care guidelines. For example, Trisomy 21, Turner syndrome, and 22q11.2 deletion syndrome all have age-based care guidelines [42,80,81,82]. There are also surveillance recommendations for patients with Trisomy 13 and Trisomy 18 [83,84]. In addition, fatty acid oxidation disorders like very long chain acyl-CoA dehydrogenase deficiency and organic acidemias, such as propionic acidemia, have specific dietary guidelines [85,86]. Furthermore, there have been several exciting treatment advancements based on genetic diagnoses, underscoring the importance of genetic evaluation in the CHD and cardiomyopathy field. 

There are new therapies available for CHD and cardiomyopathies based on genetic diagnosis. Enzyme replacement therapy can be used for certain lysosomal storage disorders. For instance, a therapy has been available for patients with Fabry disease since 2001 and for those with Pompe disease since 2006 [87,88]. Ongoing investigations are being conducted for gene therapies, and autologous hematopoietic stem cell transplants of cells treated with lentiviral vectors [87,88]. In June 2023, the Food and Drug Administration approved a therapy for Duchenne muscular dystrophy (DMD) which has the potential to impact cardiomyopathies [89]. The therapy is an adeno-associated viral vector that delivers microdystrophin for patients with premature terminations in DMD between exons 18 and 58 [89,90]. Additionally, there are multiple approved exon-skipping therapies for DMD. There are also exciting therapeutic advancements for patients with hypertrophic cardiomyopathy due to RASopathy, including the use of the MEK inhibitor trametinib [91,92,93,94,95]. 

## 6. Clinical Management Considerations

Clinical genetic evaluations should always begin with a thorough examination and history. History should start from the prenatal period for younger patients. For older patients, history should pay special attention to neurodevelopmental differences, intellectual development, school performance, medical conditions, vision or hearing concerns, growth concerns, and activity level. Any extracardiac anomalies identified on prior imaging should be noted. A physical examination should include a notation for dysmorphic features and minor anomalies, growth parameters, and a full neuromuscular assessment [3,37]. Neuromuscular evaluations are particularly critical in patients with cardiomyopathy, as many of the genetic disease-specific therapies available are for conditions that can have neuromuscular manifestations. Functional metabolic testing may be needed during evaluation to detect inborn errors of metabolism [96]. Many inborn errors of metabolism associated with cardiomyopathies also have disease-specific treatments [96]. 

When obtaining the family history, it is important to ask if any family members are known to have had any prior cardiac evaluations or testing, especially for first-degree relatives, as they may be affected and unaware. Mid-parental height can also be valuable to indicate the genetic potential for growth, as a finding of short stature can be useful in building a differential diagnosis. Having a pedigree can also help to determine which family members should receive subsequent genetic testing (cascade testing) after a patient (proband case) has been diagnosed with a pathogenic genetic variant [78]. If a pathogenic variant is discovered in a patient with inherited cardiovascular disease, the American Heart Association recommends cascade clinical and genetic testing for all first-degree relatives of the proband case [97]. This typically involves a clinical exam and diagnostic workup along with genetic testing for the specific disease-associated variant that was discovered in the patient. At-risk family members with no current clinical phenotype may still require more frequent screening and periodic surveillance for heart disease, along with modified clinical management. If a family member is found to be positive for the disease-associated variant, further cascade testing should be performed for that individual’s first-degree relatives, and cascade testing should continue until all extended family members who are at risk have been offered clinical and genetic testing [97]. Healthcare providers need to be aware of the Genetic Information Nondiscrimination Act, which is a federal law that protects against discrimination based on genetic information in health insurance and employment. However, the law does not include other types of insurance, including life and disability insurance, nor does it apply to small companies [97]. While diagnoses of CHD and/or cardiomyopathy will have an impact on insurance, regardless of genetic results, it is an important aspect to consider when testing presymptomatic family members. Providers should also be aware of any local laws and regulations regarding genetic testing [97]. An understanding of this information will help providers to answer patients’ and families’ questions.

The best approach to care for patients with CHD and cardiomyopathy utilizes a standardized, protocol-based approach to genetic evaluation and testing. These protocols should include recommendations for consultation with a medical geneticist and/or cardiovascular genetics team when available. 

Published recommendations exist for genomic evaluation in patients with CHD and cardiomyopathies, and they are regularly updated [16]. Depending on the cardiac lesion type, there may be specific, but evolving, algorithms available, with genetic testing suggestions [16]. Newer recommendations include universal genetic testing with CMA, exome sequencing, exome-based panels, and/or genome sequencing [21]. However, the chosen testing modalities depends on institutional availability. Given the rapid evolution of testing modalities and their institutional availability, there is a need for a mechanism to adjust local protocols over time. Whenever possible, it is important to ensure that the original genetic testing report is easily accessible in patients’ electronic medical records for future review, to facilitate a complete evaluation and account for the evolution of technology and medical knowledge over time. 

## 7. Summary

Genetic evaluation and testing are important components of care for patients with CHD and cardiomyopathy. The process of genetic testing is continuously evolving, and it is important to have an understanding of genetic and genomic testing modalities, along with the strengths and weaknesses of specific testing methods and the availability of testing within one’s institution. The evaluation and testing process for these patients can be complex. However, a standardized protocol-based approach has proven to be the most efficient and effective way to provide optimal care. By following this approach, healthcare providers can ensure that patients receive the best possible care and treatment.
